# Books and Authors

**Published:** 1910-01

**Authors:** 


**The Practical Medicine Series.** Ten volumes. Under the general editorial charge of **Gustavus P. Head,** M.D., Professor of Laryngology and Rhinology in the Chicago Post-Graduate Medical School. Vol. VIII. Materia Medica and Therapeutics, Preventive Medicine, and Climatology, edited by **George F. Butler,** M.D., **Henry B. Flavill,** M.D., and **Norman Bridge,** M.D. Series 1909. Chicago: The Year Book Publishers. (Price, $1.50; entire series, $10.00.)

The hints of value to be obtained on a single subject in this book more than pay the price of the entire set; for example, what may be learned of seratherapy, or of the vaccines, or of *x*-ray treatment is of exceeding usefulness to the practitioner who wants to know. Again, the section on preventive medicine contains much of interest as well of importance for every student of this question, and for all officers of the public health. Climatology is closely allied to the prevention of disease, and this topic is well handled by Norman Bridge. All of these topics contain material of late publication, and have been judiciously gleaned from the literature of the year 1908, by the heads of the several departments. We commend the volume to the general practitioner, who may purchase it separate from the others of the series if he chooses.

---

**The Practice of Medicine.** By **James Tyson,** M.D., Professor of Medicine in the University of Pennsylvania and Physician to its Hospital. Fifth edition. Octavo, pp. 1438. Illustrated. Philadelphia: P. Blakiston's Son & Co. 1909. (Cloth, $5.50.)

This is the fifth edition of this treatise since 1896, when it first appeared. Tyson is a conscientious teacher and aims to put his best endeavor into his writings. He has put his best thought into this book and has made such revisions as were needed to keep it abreast of the present knowledge concerning medical practice. It is fitting to mention the principal changes since the last edition. They include a revision of the infectious diseases, a revamping of diseases of the blood, and a freshening of the treatment of tuberculosis, with the assistance of Drs. Londeau and Baldwin, of Saranac Lake, who have helped to elaborate the tuberculosis section. Tyson states that he has given the opsonic index as much attention as it seems to deserve. He devotes something less than a page to its consideration, and says the subject is still *sub judice*—a correct estimate of its present status.

Other topics that have been enlarged or rewritten are diseases of the stomach, diseases of the circulatory system, the latter including an elaboration of the Adams-Stokes syndrome; also pericarditis has been given greater space. Tetany and exophthalmic goiter, too, have been largely rewritten, while diseases of the nervous system have been extended in some particulars.

Tyson's position in the field of internal medicine is a strong one and his treatise will continue to maintain a place in the advance line, as one of the best of the many textbooks of medical practice.

---

**A Manual of Otology.** By **Gorham Bacon**, A.M., M.D., Professor of Otology in the College of Physicians and Surgeons, Columbia University, New York. With an Introductory Chapter by **Clarence J. Blake**, M.D., Professor of Otology in the Harvard Medical School, Boston. Fifth edition, thoroughly revised. 12mo, 500 pages, 147 engravings and 12 plates. Lea & Febiger, Philadelphia and New York, 1909. (Cloth, $2.25, net.)

The favor bestowed upon this manual in the beginning still clings to it. The fifth edition is welcome as showing enterprise on the part of the author, who is ever ready to keep the book fresh. Otitis media, acute and chronic, catarrhal and purulent, has received due attention as formerly, and diseases of the mastoid process, including their complications, are handled elaborately and with skill. Bacon writes with the authority of experience; he deals with the most difficult questions in otology with skilful precision, and presents his subject in the full light of the latest knowledge pertaining to it. He has revised his book, making some additions and a few eliminations, so that this edition presents the student and practitioner with the latest and best thought concerning the otology of today.

---

**The Practical Medicine Series.** Comprising ten volumes on the year's progress in medicine and surgery, under the general charge of **Gustavus P. Head**, M.D., Professor of Laryngology and **Rhinology** at the Chicago Postgraduate Medical School. Vol. VII. Pediatrics. Edited by **Isaac A. Abt.** Orthopedic surgery. Edited by **John Ridlon.** 1909. Chicago: The Year Book Publishers. (Price, $1.25; entire series, $10.00.)

A valuable collection of excerpts has been incorporated in this volume relating to the advances of importance for the year 1908, in diseases of children and in orthopedic surgery. Diseases of the newborn are included in the first section, and the important diseases of later infancy and early childhood are also dealt with in the department of pediatrics. In the section on orthopedic surgery we find that diseases of the hip-joint, the spine, and all the larger joints are taken up, as well as infantile paralysis and a miscellaneous group of affections and injuries, all of which are important to any and every person who assumes to treat children. The book is well arranged for ready reference and is an important number of the series.

---

**A Handbook of Medical Diagnosis.** By **J. C. Wilson**, M.D., Professor of the Practice of Medicine and Clinical Medicine in Jefferson Medical College and Physician to its Hospital. Octavo, pp. 1435. Illustrated. Philadelphia and London: J. B. Lippincott Co. 1909.

Works on diagnosis are plentiful at the present time, indicating that the trend of medical science is toward perfection in the art of interpreting the symptoms of disease. To read aright the subtle manifestations of disease is the constant aim of the physician, and the nearer he approaches to exactitude in this, the greater will be his success in curing his patients. This author is familiar to the professional public through his work on practice, being a well known teacher of medicine. He is accustomed to deal with the clinical manifestations of disease and, as might be anticipated, has produced a book of great value to both student and general practitioner.

Wilson divides his work, which he is pleased to call a handbook, into four parts, making more than fourteen hundred standard octavo pages. In the first part is considered medical diagnosis in general: in the second, the methods and their immediate results; in the third, symptoms and signs; and in the fourth, the clinical applications. The author presents these several divisions in great detail, describes all important instrumental aids to diagnosis, most of which are illustrated, and gives instructions as to auscultation, palpation, posture, and every known method of detecting the signs and symptoms of disease, as well as how to interpret their relative and absolute importance.

---

**A Practical Treatise on Diseases of the Skin.** For the use of students and practitioners. By **J. Nevins Hyde**, A.M., M.D., Professor of Dermatology and Venereal Diseases in the University of Chicago, Medical Department (Rush Medical College). Eighth edition, thoroughly revised and much enlarged. Octavo of 1137 pages, with 223 engravings and 58 full-page plates, in colors and monochrome. Lea & Febiger, Philadelphia and New York, 1909. (Cloth, $5.00, leather, $6.00, net prices.)

Some of the diseases of the skin afford the most perplexing problems the general practitioner is called upon to deal with. Such a medical man should have at hand a reliable guide in the form of a treatise that is at once practical, comprehensive, and scientifically accurate. Hyde's is all of this and even more,—it is as nearly complete as it is possible to make a single volume on such a large subject. This book is increased in size over the preceding edition by 250 pages. Several new articles have been written and the entire work has been revised carefully, line by line. The treatise as it stands at present considers almost every form and variety of skin disease known to the science of dermatology. New growths, benign and malignant are dealt with comprehensively: while diseases of the skin of parasitic origin receive marked attention. Indeed, there is scarcely a skin lesion, blemish, or pathological departure from the normal that Hyde fails to note or to present in a manner that does not prove helpful to the general practitioner. Moreover, the dermatologist himself may glean many a helpful hint from its text and illustrations, while the student, too, may study the work with unfailing benefit.

We feel sure the author, who is well known as an eminent teacher in one of the largest medical institutions of the land, has succeeded in constructing one of the most satisfactory textbooks extant, improving it by careful and studious revision, as he has from time to time, and through this perfection has made the study of dermatology, heretofore repellent to the average student of medicine, a delightful, attractive topic.

---

**Renal, Ureteral, Perineal and Adrenal Tumors and Actinomycosis and Echinococcus of the Kidney.** By **Edgar Garceau,** M.D., Visiting Gynecologist to St. Elizabeth's Hospital, and to the Boston Dispensary, Boston. Octavo, pp. 434. Illustrated. New York and London: D. Appleton & Co. 1909. (Cloth, $5.00.)

Hypernephroma being the most important of growths affecting the kidney is considered first. It is, moreover, of the most frequent occurrence—more frequent indeed, than all the others if one may accept the table of the Massachusetts General Hospital, covering a period of eight years, and during which time not a single case of carcinoma was operated on. The surgery of the kidney forms a very small part of this work, it being only touched upon here and there. The chief concern of the author is with the pathology of tumors of the kidney and its adnexa, dealing also with actinomycosis and echinococcus of the kidney.

In the eleventh chapter Garceau deals with the determination of renal efficiency and here is found a most interesting and instructive setting forth of this important measure. The author states with proper emphasis that operation never should be done on a kidney unless it first be ascertained that its fellow is capable of performing the work of both; that is, it must be learned if the opposite kidney is sound. Compensatory hypertrophy, however, should not be mistaken for structural disease. The technic of cystoscopy is given in detail and several cystoscopes are described and illustrated, including the author's design, especially for use in the female.

Garceau wrote the chapter on renal tumors for the Watson-Cunningham work on genitourinary diseases recently published. Many of the illustrations employed in that chapter are used in the present treatise, as well as others from specimens in the Boston hospitals. This work will do much to clear the foggy atmosphere surrounding the pathology of tumors of the kidney, and is an intelligent exposition of the subject.

---

**A Textbook of Obstetrics.** By **Barton Cooke Hirst,** Professor of Obstetrics in the University of Pennsylvania. Sixth edition. Illustrated. Octavo, pp. 992. Philadelphia and London: W. B. Saunders Co. (Cloth, $5.00.)

Two features of this work impress one upon glancing casually at its pages. First, six editions and four reprintings have been put forth in eleven years. Second, it is filled with the anomalies to be met in practice. This latter is of special importance, the practitioner being more or less familiar with the normal in obstetrics; he has been instructed in it, and has dealt with it in the lying-in room. But the unusual, the abnormal, the irregular types of the birth canal and fetus are strange to him as a rule. In other words, Hirst deals very largely with the pathologic phenomena peculiar to women, and particularly those of the pregnant and parturient woman. This is what makes his book valuable, more valuable than works on obstetrics in general. Moreover, he tells us what to do in the presence of these abnormal or pathologic conditions, and this is precisely what the student and young practitioner want to know.

It is quite unnecessary to say that this edition has undergone thorough revision. Hirst is too experienced an author and teacher to fail in this regard. Especially have extensive additions been made in the operative sections, in which now all the operations for the complications and consequences of childbirth are described. Whenever the author recommends operative treatment, he proceeds to give the technic of the operation appropriate to the case. There is no broader view of this department of medical science than that taken by the author of this work, and it is not surprising that his treatise has reached a world-wide popularity. We regard it as one of the better of the works on obstetrics in vogue at the present time.

---

**The Physician's Pocket Account Book.** By **J. J. Taylor,** M.D., bound in full leather, 24 pages of practical instructions for physicians, 216 pages of accounts. Price, $1.00 per copy; published by the Medical Council, 4105 Walnut Street, Philadelphia, Pa.

This book is an old friend. It contains 24 pages of business instructions for physicians, which have been found useful and accurate in a long and varied experience. They are embraced under the headings of "Importance of a due bill," Fees," "Billing and collecting," "Cautions," "Statute of limitations," "Form for wills," "Dying declarations," "Saving and investing," Instant treatment of poisoning," and the like. It also contains an average fee bill which may serve as a guide in practice.

The book contains 216 pages for accounts, of which eight pages are devoted to alphabetic index, 146 pages to regular accounts, 32 pages to short accounts, 24 pages to cash accounts, and 8 pages to birth, death, and vaccination records. It is one of the best planned as well as the most compact of account books for physicians, and has only to be seen to meet with approval.

---

**The Practitioners' Visiting List** for 1910.. An invaluable pocket-sized book containing memoranda and data important for every physician, and ruled blanks for recording every detail of practice. The Weekly, Monthly and 30-Patient Perpetual contain 32 pages of data and 160 pages of classified blanks. The 60-Patient Perpetual consists of 256 pages of blanks alone. Each in one wallet-shaped book, bound in flexible leather, with flap and pocket, pencil with rubber, and calendar for two years. Price by mail, postpaid, to any address, $1.25. Thumb-letter index, 25 cents extra. Descriptive circular showing the several styles sent on request. Lea & Febiger, Publishers, Philadelphia and New York.

This excellent visiting list comes to hand with promptitude and is equal to its traditions in appearance and improvement. The description given in the title as printed in the heading does but scant justice to this beautiful book, which is one of the most useful of its kind. The present edition contains among other valuable information a scheme of dentition; tables of weights and measures and comparative scales; instructions for examining the urine; diagnostic table of eruptive fevers; incompatibles, poisons and antidotes; directions for effecting artificial respiration; extensive table of doses; an alphabetical table of diseases and their remedies, and directions for ligation of arteries. The record portion contains ruled blanks of various kinds, adapted for noting all details of practice and professional business.

---

**Diseases of Children.** By **Henry Enos Tuley,** M.D., Professor of Obstetrics, University of Louisville. Octavo, pp. 662. Illustrated. Baltimore: Southern Publishing Co. 1909. (Cloth, $5.00; half leather, $6.00.)

A work on diseases of children appearing at this time comes to an already somewhat overstocked market, hence must depend on its merit for success. After a reasonably careful examination of this book we feel sure it will obtain, at the hands of the general practitioner, a large measure of favor; certainly it deserves it. The author is a teacher of experience and a clinician of precision.

In the first chapter, Tuley describes the anatomical differences between the infant and the adult, then deals with the care of the newborn. His directions are wholesome and are worthy of careful observation in the lying-in room. We approve of his advice regarding Credé's management of the eyes of the newborn, but think so important a matter should receive even more attention. In view of the work done by the committee of the American Medical Association, of which Dr. F. Park Lewis, of Buffalo, is chairman, which has shown that 30 per cent. of existing blindness is due to ophthalmia neonatorium and therefore preventable, we think it behooves every author, who feels called upon to deal with the subject at all, to handle it without gloves and in detail. Credé's directions, at the very least, should be given in full with minutest particulars.

Tuley pays considerable attention to infant feeding and his directions are to be commended. He illustrates a "certified barn" and a tuberculous cow which are useful object lessons. In handling the diseases of infancy and childhood, general and special, the author is deserving of commendation for his thoroughly scientific methods. His therapeutics are beyond criticism and his diagnostic acumen is of the first order. The treatise is not only a credit to its accomplished author but is a valuable addition to the literature of pediatrics.

**Surgical Diagnosis.** By **Daniel N. Eisendrath,** M.D., Professor of Surgery in the Medical Department of the University of Illinois (College of Physicians and Surgeons). Second revised edition. Octavo of 885 pages, with 574 original illustrations, 25 in colors. Philadelphia and London: W. B. Saunders Company. 1909. (Cloth, $6.50; half morocco, $8.00, net prices.)

When the first edition of this great work appeared we expressed the opinion that a place for it existed, and we now wish to state that such a treatise was needed. A second edition being called for within two years would seem to justify this statement. The author, seeking to keep his treatise well abreast of the science, not only has revised the text of the previous edition but has added new material to a considerable extent, so it may be said of this edition that it represents the last thought relating to surgical diagnosis. Especially may we mention the section on diagnosis of renal lesions, which has been rewritten to include the advances so rapidly taking place in this important field. We wish to mention in particular the illustrations all through the book, which are of such high class, mostly original, and some in colors. The treatise is so original in conception and design that it bespeaks the scientific skill and knowledge of its author from the first to the last page.

---

**The Medical Complications, Accidents and Sequels of Typhoid Fever and the Other Exanthemata.** By **H. A. Hare,** M.D., B.Sc., Professor of Therapeutics in the Jefferson Medical College, and **E. J. G. Beardsley,** M.D., L.R.C.P., Philadelphia. With a special chapter on the Mental Disturbances Following Typhoid Fever, by **F. X. Dercum,** M.D., Professor of Nervous Diseases in the Jefferson Medical College. Second edition. Octavo, 398 pages, with 26 engravings and 2 plates. Lea & Febiger, Philadelphia and New York, 1909. (Cloth, $3.25, net.)

No disease, perhaps, in the whole range of human maladies, is more frequently met by the general practitioner than typhoid fever; hence, it behooves him to be prepared to deal with it not only in its typical but in its aberrant forms. Hare's essay, as he is pleased to call it, deals with the complications and sequels of this and the other exanthemata. In this edition Dr. E. J. G. Beardsley becomes associate author, and together they have completely revised the book to include the very latest knowledge pertaining to these affections. Professor F. X. Dercum supplies a chapter on mental disturbances complicating or following typhoid fever, making the work quite complete in all its details. It is important to prevent or arrest the sequels, before they have proceeded to the danger point. It is not always possible to do this, but in this book will be found valuable hints in that direction.

It has been said, and with much truth, the period of convalescence is the most dangerous time in the disease. Hare devotes a chapter to the complications of this period, from which many valuable lessons may be learned. Perhaps most puzzling of all features of typhoid, most difficult to comprehend, are the aberrant forms of the disease, those in which all the classic manifestations are so masked that they cannot be discerned or properly mapped out. It is in the discussion of these that Hare's book possesses special interest and value. It is a work that stands alone and appeals to every physician who visits the bedside of the sick.

**A Treatise on Diseases of the Nose, Throat and Ear.** By **William Lincoln Ballenger,** M.D., Professor of Laryngology, Rhinology and Otology in the College of Physicians and Surgeons, Chicago. New (2) edition, thoroughly revised. Octavo, 930 pages, with 491 engravings, mostly original, and 17 colored plates. Lea & Febiger, Philadelphia and New York. 1909. (Cloth, $5.50, net.)

To an author whose first edition is exhausted within a year after its appearance, it must be gratifying to be asked for a revision at once. Ballenger calls it an "unexpected opportunity," which is a modest, earnest expression of appreciation. He then proceeds to state that he has scrutinised the text of his first edition line by line, in order that clearness of expression may be obtained, correcting every page that needed it, rewriting many of them, adding new material here and there, thus bringing the entire work forward to the last moment of its printing. Some of the chapters have been considerably enlarged, notably those on the surgery of the nasal accessory sinuses and on the surgery of the tonsils.

The section on the ear has been improved, placing it on a plane of satisfaction to every otologist. The book as it now stands may be fairly regarded as the most modern exposition of diseases of the nose, throat and ear by an American author. The illustrations, mostly original, are of a high class and exhibit the artistic and anatomic knowledge of the author. The treatise meets the requirements of student, teacher, and practitioners. Since the work in its first edition, printed from the manuscript, immediately rose to the foremost place in its literature, it may be safely predicted that this revision, starting with the advantage of a printed book as a basis, will achieve still wider success. It is an unrivaled source of practical information.

